# Regulation of Social Recognition Memory in the Hippocampal Circuits

**DOI:** 10.3389/fncir.2022.839931

**Published:** 2022-03-30

**Authors:** Xinnian Wang, Yang Zhan

**Affiliations:** ^1^Shenzhen Institutes of Advanced Technology, Chinese Academy of Sciences (CAS), Shenzhen, China; ^2^Division of Life Sciences and Medicine, School of Life Sciences, University of Science and Technology of China, Hefei, China; ^3^Guangdong Provincial Key Laboratory of Brain Connectome and Behavior, CAS Key Laboratory of Brain Connectome and Manipulation, Shenzhen Institutes of Advanced Technology, Chinese Academy of Sciences, Shenzhen-Hong Kong Institute of Brain Science-Shenzhen Fundamental Research Institutions, Shenzhen, China

**Keywords:** social recognition memory, dorsal CA2, ventral CA1, social isolation, oxytocin

## Abstract

Social recognition memory reflects the ability of the social animals to recognize and remember familiar individuals of the same species. The unique ability for mammals to recognize conspecifics is essential and beneficial when animals conduct daily social activities. This brief review summarizes a brain network underlying social recognition memory and explores the possible relationships between social isolation and social recognition memory. Finally, we introduce some possible related molecular mechanisms underlying social recognition memory. These findings help us explore potential targeting brain areas or circuits of social communication disorder.

## Social Recognition Memory

For animals living in social groups, only individuals with social recognition memory can correctly respond to the visitors in cooperative or competitive social situations, such as remembering their mating partner (Okuyama et al., 2014; Walum and Young, 2018) and recognizing threatening visitors to the animal’s habitats (Jacobs et al., 2016). If social animals cannot remember familiar conspecifics, the stability of the social group will be destroyed. We focus on the ability of the animal to recognize individual partners during the interaction between the two parties. Behavioral paradigms which can quantitatively test the recognition ability are described and discussed.

### Individual Recognition

**FIGURE 1:**
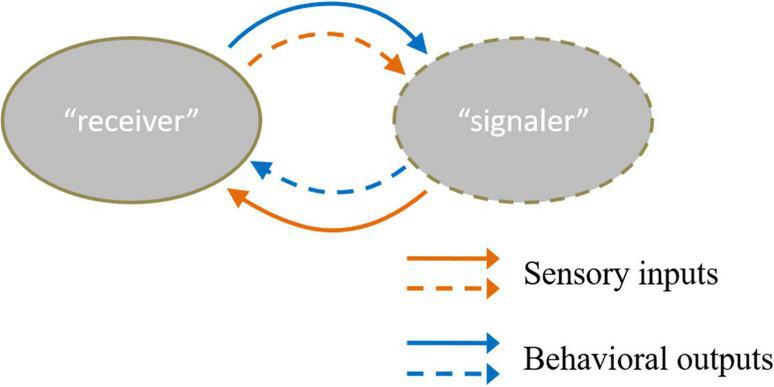
There are two kinds of sensory inputs and behavioral outputs between “receiver” and “signaler” (Chen and Hong, 2018). The solid line means the sensory cues “receiver” received and the behavior that the “receiver” conducts. Moreover, the dotted line refers to the sensory cues “signaler” received and the behavior the “receiver” acts. The orange color line describes the input and output information of the “receiver”. The blue color line referred to input and output information of the “signaler”.

Individual recognition refers to a subset of recognition that occurs when one organism recognizes another based on its unique characteristics (Dale et al., 2001). Almost all social behaviors require individual recognition. In individual recognition, the recognizer is regarded as “receiver,” and the sending individual is recognized as “signaler” (Tibbetts and Dale, 2007). Most studies focus on “receiver,” and relatively few have explored “signaler.” In fact, individual recognition is a task that requires cooperation between the “receiver” and “signaler.” The two parties can form a reciprocal feedback loop between them to interact with each other (Chen and Hong, 2018). The information from acute sensory inputs detected from the “signaler” is transformed into the behavioral output of the “receiver.” In turn, the behavioral output of the “receiver” also provides sensory cues to the “signaler” (Figure 1).

### Social Recognition Memory Paradigms

In rodents, mice and rats tend to investigate the novel social stimulus with longer investigation compared to familiar social stimulus. This innate tendency reflects a marked difference in individual recognition for these two social stimuli. Social recognition memory can be studied by detecting the investigation time of different social stimuli.

**FIGURE 2:**
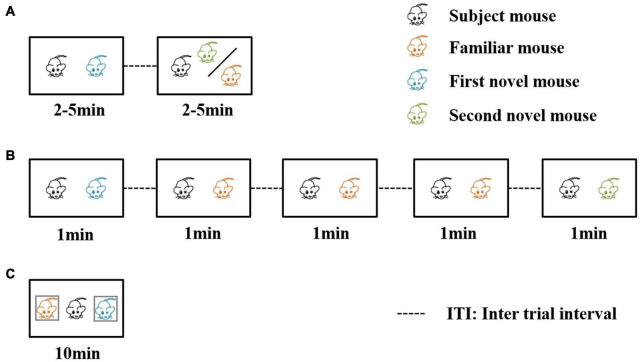
Three behavioral paradigms for evaluating social recognition memory. In the first two of the three paradigms, a familiar mouse means the social stimulus the subject mouse has met in the previous trials. In the third paradigm, a familiar mouse refers to a cage mate of the subject mouse. **(A)** The direct interaction paradigm includes two encounters. The social stimulus the subject mouse meets in the second encounter can be familiar or novel. **(B)** The social habituation-dishabituation paradigm includes five trials of encounters. In the fifth trial, the subject mouse meets a novel social stimulus. **(C)** The social discrimination paradigm includes one encounter trial. The subject mouse meets two social stimuli at the same time. One is a familiar cage mate, and the other is a novel stimulus.

Three well-established behavioral paradigm methods are used to evaluate social recognition memory (Leser and Wagner, 2015; Figure 2). The first paradigm is the two-trial social recognition (Figure 2A). The receiver animal first has an encounter with a social stimulus. Then in the next trial, the receiver animal encounters a familiar stimulus that has been presented in the first trial or another novel social stimulus that has never been encountered. Reduced investigation time toward the familiar stimuli reflects the establishment of social recognition memory (Thor and Holloway, 1982; Thor et al., 1982; Kogan et al., 2000). A series of time intervals can be set between the two encounter trials, including immediate, 30 min, 24 h, three days, and seven days. The ability to recognize the familiar stimuli after the encounter of the first social stimuli with different inter-trial intervals between the two trials can be used to define short-term or long-term social memory (Kogan et al., 2000). An interval being intermediate or 30 min is regarded as short-term social memory, and intervals being 24 h, three days, or seven days are regarded as long social recognition memory. The advantage of this paradigm is that definite and visible different types of social behavior can be studied, such as sniffing, chasing, and allogrooming. The limitation of this paradigm is the aggressive behavior between the receiver animal and the social stimuli. To avoid the fighting situation, three-chamber social interaction is also frequently used. In this paradigm, the social stimuli are confined to a small cup with wires or bars so the receiver animal can not only freely explore the cup containing the social stimuli but also make real contacts (Moy et al., 2004). Recent studies also included two stimulus mice contained in the cup for the first encounter trial (Oliva et al., 2020), and in the recognition trial one of the stimuli was replaced by a novel one.

The second paradigm is the social habituation-dishabituation paradigm (Figure 2B). This paradigm consists of five trials of encounters (Sekiguchi et al., 1991). The first four encounters are the same social stimulus and the last one is a novel social stimulus. The receiver animal displays a reduction of investigation time, and when the novel social stimulus is presented, the investigation time increases. The paradigm has the advantage of an obvious establishment for social recognition memory. The investigation time shows a continuous reduction during repeated exposure of the first social stimulus and there was a strong recovery of investigation of the novel social stimulus in the last trial.

The third one is the social discrimination paradigm (Figure 2C). This paradigm includes an encounter in the situation that the familiar stimulus and the novel stimulus are presented to the receiver simultaneously (Engelmann et al., 1995). Usually, the two social stimuli are confined to small cups so that the receiver animal can decide to explore either cup containing the stimulus animals. A longer investigation of the novel social stimulus indicates the establishment of social recognition memory. In the third paradigm, a familiar mouse refers to a littermate of the tested mouse or a mouse that experienced the familiarization phase for at least 2 h. There is a separate time for the receiver animal, and the mouse experienced the familiarization phase. Okuyama et al. (2016) found that different intervals of separation time can also affect the social memory as a long-term duration of more than 24 h can lead to failure to recognize the two social stimuli.

## Brain Network Underlying Social Recognition Memory

**FIGURE 3:**
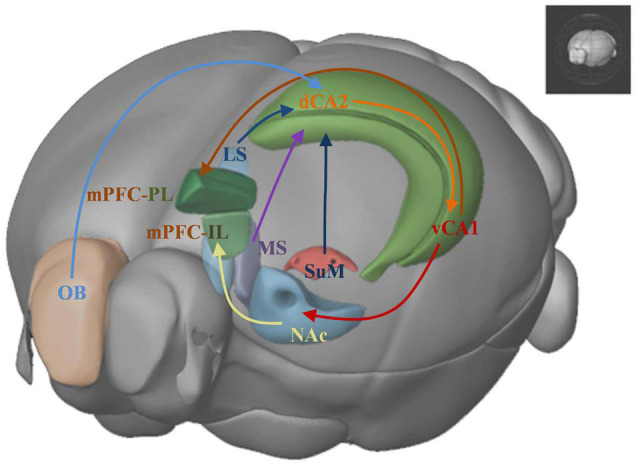
Brain networks underlying social recognition memory. OB: olfactory bulb; dHPC: dorsal hippocampus; dCA2: dorsal CA2; LS: lateral srptum; MS: medial septum; SuM: supramammillary nucleus; vCA1: ventral CA1; NAc: nucleus accumbens; IL: infralimbic area; PL: prelimbic area; mPFC: medial prefrontal cortex.

Social cognitive neuroscience studies mental, behavioral, and neural mechanisms underlying social behavior in socially organized individuals (Ochsner and Lieberman, 2001). It aims to understand the reasons and the processes of social interactions between individuals. Three levels of research have been described to understand the social behavior, the social level, the cognitive level and the neural level. Recent studies combine the tools from the three levels to uncover the brain mechanisms underlie the social behavior and the information-processing principles responsible for different forms of social behavior. Here we primarily introduce a brain network (Figure 3) which focus on the neural level to understand relevant brain areas that regulate social recognition memory.

### Hippocampus

Early lesion studies have shown that lesioning of the entire hippocampus can affect social memory, as detected by the direct social recognition memory paradigm described in Figure 1A (Kogan et al., 2000). Bilateral microinjection of ibotenic acid in the hippocampus disrupted the social recognition memory when the second trial was performed 30 min after the first encounter trial. However, if the second trial was performed immediately after the first trial, the social recognition memory was not affected. This experiment demonstrates that lesioning the hippocampus can cause social memory deficit but social recognition between two close encounters of familiar and novel stimuli may not require the hippocampus. Using protein synthesis inhibitor anisomycin, blocking the protein synthesis in the hippocampus did not impair the 24-h interval test of social recognition memory. However, the protein inhibitor did not affect the social recognition memory using a 30 min interval. The cyclic AMP responsive element-binding protein (CREB) mutant mice, lacking isoforms of CREB, could not form long-term social recognition memory but could form short-term social recognition memory. The results with the protein inhibitor and the CREB mutant mice demonstrate that intact hippocampal functions are required for long-term social recognition memory.

### Hippocampal Subregions

Hippocampal lesioning studies point to a role of the hippocampus in the social recognition memory, however, this approach can not disclose which subregions of the hippocampus contribute to the social recognition memory. It has been found that the mRNA level of vasopressin 1b receptor is higher in the CA2 subregion (Young et al., 2006) and complete deletion of this receptor impaired social recognition memory (Wersinger et al., 2002; DeVito et al., 2009). Targeting the CA2 subregion using excitotoxic N-methyl-D-aspartate (NMDA) to silence CA2 region caused impairment in the social recognition memory in three-chamber social recognition and habituation-dishabituation social memory tests (Stevenson and Caldwell, 2014).

Taking advantage of transgenic mouse line Amigo2-cre in which cre is selectively expressed in the pyramidal cells of the dorsal CA2 region, it was found that inactivation of the pyramidal neurons in this region impaired social recognition memory in three-chamber social recognition and 5-trial habituation-dishabituation social memory tests (Hitti and Siegelbaum, 2014). There was no change for social preference or several other non-social memories. Additionally, pharmacogenetic and optogenetic approaches to inhibit dorsal CA2 acutely and reversibly have revealed that dorsal CA2 was involved in all the phases of encoding, consolidation, and retrieval of social recognition memory (Meira et al., 2018). These results demonstrate that dorsal CA2 plays an essential role in social recognition memory.

The ventral hippocampus region is regarded to have a role in emotional processing such as anxiety and fear memory (Fanselow and Dong, 2010). Recently subregion ventral CA1 has been found to regulate social recognition memory. Okuyama et al. found that social memory was stored in ventral CA1 neurons (Okuyama et al., 2016). They discovered that optogenetic inhibition of the excitatory neurons in the ventral hippocampus but not in the dorsal hippocampus resulted in a deficit in social recognition memory. Using cfos transgenic animals and channelrhodopsin tools to label the ventral CA1 neurons that were specifically activated by the social stimuli, the social discrimination test failed to detect the establishment of social recognition memory 24 h after the first encounter, and furthermore optogenetic activation of the labeled neurons could restore the memory. These results indicate that there existed neurons in the ventral CA1 that could store the social memory.

### Afferent and Efferent Areas of the Hippocampus

The afferent and efferent connectivity patterns of the hippocampus change along the longitudinal axis (Strange et al., 2014). Therefore, given the roles of the dorsal and ventral hippocampus in social recognition memory, their afferent and efferent pathways may also contribute to the regulation of the social recognition memory. Interestingly, it has been described that within the hippocampus dorsal CA2 neurons provide excitatory inputs to ventral CA1 pyramidal neurons. This indicates that these two hippocampal subregions may interact with each other to process social cues involved in the social recognition memory. Using pharmacogenetic tools, inhibition of the dorsal CA2 to ventral CA1 pathway caused the receiver subject unable to recognize the familiar conspecific (Meira et al., 2018). Chen et al. (2020) discovered that the supramammillary nucleus (SuM) to dorsal CA2 circuit took part in recognizing social novelty. When the pathway SuM to dorsal CA2 was optogenetically activated, the social recognition memory was impaired, and the mice showed a higher exploration time with familiar social stimulus. The medial septum (MS) was a dorsal CA2 afferent area critical to social memory (Wu et al., 2021). Pharmacogenetic inhibition of the MS to dorsal CA2 circuit impaired social recognition memory (Wu et al., 2021).

The afferent and efferent pathways of the ventral hippocampus also contributed to the process of social recognition. Optogenetic activation of the neural circuit of ventral CA1 to nucleus accumbens (NAc) shell reduced the time for the receiver subject to explore the novel stimulus in the social discrimination task (Okuyama et al., 2016). Interestingly, when subject mice were exploring familiar littermates, optogenetic inhibition of the ventral CA1 to NAc circuit also impaired social recognition memory. Phillips et al. (2019) found that ventral hippocampus to the medial prefrontal cortex projection was overactive in Mecp2 knockout mice. The long-term excitement of medial prefrontal cortex (mPFC)-projecting ventral hippocampal neurons in wild-type mice impaired social memory, and their inhibition of these neurons in Mecp2 mice rescued social memory deficits.

### Other Brain Areas Underlying Social Recognition Memory

In addition to the hippocampal circuits mentioned above, Tanimizu et al. (2017) found higher cfos and Arc expression level in the medial prefrontal cortex (mPFC), the anterior cingulate cortex (ACC), and the amygdala (basolateral area, BLA), when the subject animals were subjected to the social recognition memory tasks. Microinjecting protein synthesis inhibitors after the first encounter with the stimulus into these brain areas, respectively, showed that protein synthesis of these brain regions is required for long-term social recognition memory but not short-term social recognition memory. The genetic and pharmacological disruption of glutamatergic synaptic transmission in ventral rather than dorsal CA3 suggests that ventral CA3 may be required for encoding social memory (Chiang et al., 2018). The lack of vasopressin receptors in lateral septum also led to impaired social recognition memory (Bielsky et al., 2005). Wu et al. (2021) discovered that serotonin (5-HT) modulation on MS bidirectionally regulated social recognition memory formation. Both pharmacogenetic and optogenetic suppression of the MS disturbed social recognition memory. Interestingly, in an autistic mouse model (Neuroligins mutant mice) with social memory deficits, injecting 5-HT receptor agonists into MS prolonged the duration spent with novel social stimulus and rescued the original social memory deficits. This 5-HT related neural circuit expands researchers’ understanding of the neural mechanisms in social memory.

Lateral septum has been implicated in the social behavior (Menon et al., 2022). Bielsky et al. (2005) found that blocking the functions of the vasopressin 1a receptors in lateral septum (LS) led to impaired social recognition memory using social habituation-dishabituation and social discrimination tasks. The social recognition memory deficits could be restored when the vasopressin receptors were re-expressed (Bielsky et al., 2005). A recent study using pharmacogenetic approach to target the oxytocin receptor expressing neurons (Oxtr-cre mice) has found that activation of these neurons could improve social recognition deficit in animal models with social deficits induced by the valproic acid (Horiai et al., 2020). Using another genetic model with neuroligin3 gene mutation, pharmacogenetic activation of LS neurons expressing the oxytocin receptors could also rescue the social recognition deficits. In Table 1, we summarized different social recognition memory paradigms and other non-social recognition tasks that were employed to test the functions of different brain areas. Inhibition of parvalbumin interneurons in the ventral CA1 led to the impairment of both social recognition memory and object recognition. Manipulation of other brain areas or circuits only affected the social recognition memory but had no effects on the object recognition (Table 1).

**TABLE 1:** Behavioral outcomes after manipulation of neural circuits using different social and non-social behavioral paradigms.

Behavior	Manipulation in cited articles	Social recognition					Object recognition	Olfaction test	
				
Article		Two-trial	Three Chamber	Five-trial	One-trial	Resident-intruder	Novel object recognition	Olfactory habituation/dishabituation	Buried food
Kogan et al. (2000)	Whole HPC lesion	+	−	−	−	−	−	−	−
Hitti and Siegelbaum (2014)	dCA2 inhibition	+	+	+	−	−	+ ^(*)^	+	+
Stevenson and Caldwell (2014)	CA2 silence	+	−	+	−	−	−	+	+
Meira et al. (2018)	dCA2, dCA2-vCA1 inhibition	+	+	−	−	−	−	−	−
Okuyama et al. (2016)	vCA1, vCA1-NAc inhibition	−	−	−	+	+	+^(*)^	−	−
Deng et al. (2019)	PVIs inhibition in vCA1 PVIs activation in vCA1	−	+	−	+	−	+ ^(*Ns*)^	−	−
Phillips et al. (2019)	vHPC-mPFC inhibition in WT vHPC-mPFC inhibition in Mecp2	−	+	−	+	−	+^(*)^	−	−
Chen et al. (2020)	SuM-dCA2 activation	+	−	+	−	−	−	−	−
Wu et al. (2021)	MS, MS- dCA2 inhibition	+	+	+	−	−	+^(*)^	−	−
Tanimizu et al. (2017)	Protein synthesis inhibition of mPFC, ACC, BLA	+	−	−	−	−	−	−	−
Sun et al. (2020)	PVIs activation in mPFC	−	−	−	+	−	+^(*)^	−	−
Almeida-Santos et al. (2019)	OB, dHPC inhibition	+	−	−	−	−	−	−	−
Park et al. (2021)	NAc-IL inhibition NAc-IL activation	−	+	−	−	−	+^(*)^	−	−
Ferguson et al. (2000)	Oxytocin knockout mice	+	−	+	−	−	−	+	−
Lee et al. (2008)	OXTRs knockout mice	+	−	+	−	−	−	−	−
Bielsky et al. (2005)	VAPRs knockout mice	+	−	+	−	−	−	+	−
Ferguson et al. (2002)	Oxytocin and vasopressin treatment	+	−	+	−	−	−	−	−
Raam et al. (2017)	Viral recombination of OXTRs in anterior DG, CA2, CA3	−	−	−	+	−	+^(*)^	−	−
Lin et al. (2018)	Conditional deletion of the OXTRs in CA2/CA3	−	+	+	−	−	−	−	−
Chiang et al. (2018)	vCA3 inhibition	+	+	−	−	−	+^(*)^	−	−

*+Behavior performed –Behavior not performed. Two-trial: Two-trial social recognition paradigm in Figure 2A. Five-trial: Social habituation-dishabituation paradigm in Figure 2B. One-trial: Social discrimination paradigm in Figure 2C.*

*Represents that both experimental and control groups have a significant difference between the investigation time of the novel object and the familiar object, indicating the intact object recognition memory. Ns means that experimental groups have no difference between the investigation of the novel object and the familiar object while the object recognition memory in the control groups remained unaffected, indicating the impaired object recognition memory. PVIs: Parvalbumin (PV) interneurons; Oxytocin receptors: OXTRs; Vasopressin 1a receptors: VAPRs.

### Possible Neuron Types Regulating the Social Recognition Memory

Different neuronal types in the subregions of the hippocampus may have differential roles in social recognition memory. Single-unit recordings from the dorsal hippocampus in rats showed that the spiking activities did not distinguish between different individuals, but they could distinguish between rats and inanimate objects (von Heimendahl et al., 2012). There existed a small number of neurons that could respond to other conspecifics. Rao et al. (2019) found that ventral CA1 neurons in rats had a strong response to the presence of conspecifics, while the response to non-social objects was almost none. These data demonstrate that at the individual neuron level the hippocampus could have a differential response to different social stimuli.

Parvalbumin (PV) interneurons in the ventral hippocampus also played a role in social recognition. Using fiber photometry, the calcium activity of PV neurons in the ventral CA1 was higher when the subject mice approached novel mice compared to the calcium activity when the subject mice approached familiar mice (Deng et al., 2019). Functionally removing PV interneurons from the ventral CA1 led to social recognition deficits. Optogenetic activation of PV interneurons only interrupted social memory retrieval but did not interrupt encoding or consolidation (Deng et al., 2019). These findings indicate that interneurons in the ventral CA1 contribute to the social recognition memory.

In addition to the neuron type in the hippocampus which can regulate the social recognition memory, different neuron types in other areas that have connections with the hippocampus also play important roles. Increased synaptic strength of the ventral hippocampal input to the fifth layer of mPFC pyramidal neurons was determined to be a factor of social memory deficits in Mecp2 mice (Phillips et al., 2019). The investigation of the novel social stimulus increased the activity of MS neurons projecting to dorsal CA2 and induced the glutamatergic synaptic plasticity in this MS-dorsal CA2 pathway (Wu et al., 2021). Futhermore, inhibitory neurons in the projection target area can also regulate social recognition memory. Sun et al. discovered that optogenetic activation of PV neurons in mPFC but not somatostatin-positive (SST) neurons could rescue the social recognition memory impairment induced by the inhibition of the ventral hippocampus (Sun et al., 2020).

## The Effect of Social Isolation on Social Recognition Memory

The absence of companions may endanger the mental health of social animals. Thor and Holloway reported that the rats that experienced social isolation had impaired social recognition memory (Thor and Holloway, 1982). However, even the 24-h social isolation in mice and rats could result in impaired long-term social recognition memory (Kogan et al., 2000). Impaired social recognition memory is also associated with altered hippocampal functions. Compared with group-housed mice, socially isolated mice during their infancy had fewer PV interneurons in the ventral hippocampus and exhibited impaired social recognition memory (Deng et al., 2019). Almeida-Santos et al. divided the adult male mice into two groups, one group was raised in a group-housed condition, and the other one was raised separately for seven days in socially isolated condition. Social isolation increased the release of glutamate in the olfactory bulb, while glutamate release in the dorsal hippocampus was decreased (Almeida-Santos et al., 2019). Blocking AMPA or NMDA receptors in the olfactory bulb or injecting AMPA into the dorsal hippocampus could restore impaired long-term social recognition memory. These results indicate that social isolation may impair the normal glutamatergic signals in the dorsal hippocampus that is required for social recognition memory.

Interestingly, the social recognition memory deficit induced by the social isolation of the adult mice can be recovered by group housing for more than a few weeks (Park et al., 2021). However, the post-weaned juvenile mice that were singly housed for eight weeks still suffered social recognition memory deficits after they were group-housed for four weeks for re-socialization (Park et al., 2021). When group-housed mice encountered the familiar conspecifics, neurons in the infralimbic area of the prefrontal cortex (IL) projecting to the NAc shell showed activation. The activation was not observed in the isolated mice. Pharmacogenetic inhibition of these neurons in wild-type mice can impair social recognition memory without affecting social preference. Activation of these projecting neurons could reverse the social recognition memory deficits in the isolated mice. These results demonstrated that early social experience can affect the social recognition memory in adulthood, possibly with the involvement of the prefrontal to the NAc pathway.

## The Molecular Mechanism Underlying Social Recognition Memory

The molecular components within the neural circuits provide insights into how the neurotransmitters or neuropeotide can contribute to the modulation of social recognition memory. It has been reported that oxytocin knockout mice had social recognition memory deficits. These mice investigated the familiar stimulus and the novel one without a clear preference, however, they did not have damaged novelty recognition ability (Ferguson et al., 2000). Similar results were also found in oxytocin receptor knockout mice (Lee et al., 2008). Vasopressin 1a receptor knockout mice also displayed deficits in social recognition memory (Bielsky and Young, 2004; Bielsky et al., 2004). The oxytocin transmission in the LS appear to the important for the social recognition. Here we focus the oxytocin functions in the hippocampal regions.

It has been hypothesized that oxytocin and vasopressin play differential roles in social recognition memory (Ferguson et al., 2002). Since oxytocin given before but not after the initial encounter in the five-trial habituation-dishabituation social memory test restored social recognition in oxytocin knockout mice, oxytocin appears critical for the acquisition rather than the consolidation phase of memory (Ferguson et al., 2000). As vasopressin facilitates recall when given after an initial encounter, vasopressin appears important for the consolidation, not for the acquisition of social memory (Ferguson et al., 2002).

Given the involvement of the dorsal CA2 in the social recognition memory, oxytocin pathways in this subregion of the hippocampus appear to be important for social recognition memory. Oxytocin receptors were expressed in hippocampal CA2 and CA3 pyramidal neurons (Raam et al., 2017). Conditional deletion of the oxytocin receptors in the CA2/CA3 region impaired social recognition memory. Optogenetic activation of the CA2/CA3 outputs in the posterior CA1 area impaired the social recognition memory. Lin et al. studied the synaptic transmission in the CA2/CA3 region-specific oxytocin receptor knockout mice and they found that the entorhinal cortex to CA2 synaptic transmission was impaired (Lin et al., 2018). These results point to the role of the oxytocin pathways in the social recognition memory.

## Conclusion

Social recognition memory is critical for distinguishing between familiar and novel individuals and it forms an essential and basic part of social behavior. This brief review first summarizes the brain regions and neural circuits related to social recognition memory, mainly dorsal CA2 and ventral CA1, and their upstream and downstream circuits, including medium septum (MS), supramammillary nucleus (SuM), lateral septum (LS), prefrontal cortex and nuculeus accumbens (NAc). Then we discuss the neuron types within the hippocampal subregions underlying social recognition memory. Although both the dorsal and ventral hippocampus regions are involved in the social recognition memory, these two regions have very different input and output brain areas. It remains unknown whether there are functionally dissociable neurons that have connections with the dorsal and ventral hippocampus to regulate social recognition memory. These are the open hypotheses that will be explored further. Social isolation can lead to impaired social recognition memory in rodents. Consequently, the neural circuit mechanism underlying social isolation may be instructive to the understanding of the neural circuits that regulate social recognition memory. Re-socialization by group-housing conditions could ameliorate the social recognition memory deficits in the socially isolated adult mice. Finally, we investigated the contribution of oxytocin pathways in social recognition memory, with a particular focus on the oxytocin transmissions in the hippocampus. Still many unknown neural mechanisms at multiple levels about why and how animals recognize individuals. Future work needs to employ experimental methods and tools at behavioral, circuitry, and molecular levels to underpin our understandings of the social recognition memory.

## Author Contributions

Both authors discussed the ideas, wrote the manuscript and edited the manuscript.

## Conflict of Interest

The authors declare that the research was conducted in the absence of any commercial or financial relationships that could be construed as a potential conflict of interest.

## Publisher’s Note

All claims expressed in this article are solely those of the authors and do not necessarily represent those of their affiliated organizations, or those of the publisher, the editors and the reviewers. Any product that may be evaluated in this article, or claim that may be made by its manufacturer, is not guaranteed or endorsed by the publisher.
